# The Protective Effects of the Proteasome Inhibitor Bortezomib (Velcade) on Ischemia-Reperfusion Injury in the Rat Retina

**DOI:** 10.1371/journal.pone.0064262

**Published:** 2013-05-14

**Authors:** Fang-Ting Chen, Chung-May Yang, Chang-Hao Yang

**Affiliations:** 1 Department of Ophthalmology, Far Eastern Memorial Hospital, Ban-Chiao, Taipei, Taiwan; 2 Department of Ophthalmology, National Taiwan University Hospital, Taipei, Taiwan; 3 Department of Ophthalmology, National Taiwan University College of Medicine, Taipei, Taiwan; UAE University, Faculty of Medicine & Health Sciences, United Arab Emirates

## Abstract

**Purpose:**

To evaluate the protective effects of bortezomib (Velcade) on ischemia-reperfusion (IR) injury in the rat retina.

**Methods:**

The rats were randomized to receive treatment with saline, low-dose bortezomib (0.05 mg/kg), or high-dose bortezomib (0.2 mg/kg) before the induction of IR injury. Electroretinography (ERG) was used to assess functional changes in the retina. The expression of inflammatory mediators (iNOS, ICAM-1, MCP-1, TNF-α), anti-oxidant proteins (heme oxygenase, thioredoxin, peroxiredoxin), and pro-apoptotic proteins (p53, bax) were quantified by PCR and western blot analysis. An immunofluorescence study was performed to detect the expression of iNOS, oxidative markers (nitrotyrosine, 8-OHdG, acrolein), NF-κB p65, and CD 68. Apoptosis of retinal cells was labeled with *in situ* TUNEL staining. Neu-N staining was performed in the flat-mounted retina to evaluate the density of retinal ganglion cells.

**Results:**

ERG showed a decreased b-wave after IR injury, and pretreatment with bortezomib, especially the high dosage, reduced the functional impairment. Bortezomib successfully reduced the elevation of inflammatory mediators, anti-oxidant proteins, pro-apoptotic proteins and oxidative markers after IR insult in a dose-dependent manner. In a similar fashion, NF-κB p65- and CD 68-positive cells were decreased by bortezomib treatment. Retinal cell apoptosis in each layer was attenuated by bortezomib. The retinal ganglion cell density was markedly decreased in the saline and low-dose bortezomib groups but was not significantly changed in the high-dose bortezomib group.

**Conclusions:**

Bortezomib had a neuro-protective effect in retinal IR injury, possibly by inhibiting the activation of NF-κB related to IR insult and reducing the inflammatory signals and oxidative stress in the retina.

## Introduction

Ischemia-reperfusion (IR) injury is caused by transient disruption of blood supply in tissues followed by reperfusion. This phenomenon contributes to various clinical problems after stroke, myocardial infarction, shock, and organ transplantation. Emerging evidence suggests that IR injury is also implicated in the pathogenesis of several vision-threatening ophthalmic disorders, such as retinal vascular occlusive disease, glaucoma and diabetic retinopathy [1]–[3]. The postulated pathophysiologic mechanisms of ischemic cell injury include depletion of adenosine triphosphate (ATP) and disturbance of intracellular calcium homeostasis, which results in cell death [4]. Furthermore, the reperfusion status aggravates the tissue insult because of released reactive oxygen species and proinflammatory mediators, which recruit inflammatory cells into tissues [5]–[7].

The retinal ischemia-reperfusion (IR) state of a rat can be accomplished by increasing the intraocular pressure through cannulation of the eye to interfere with retinal circulation followed by natural reperfusion [8], [9]. Electroretinography (ERG) showed a decreased a-wave and b-wave after the ischemic event, with the b-wave predominantly affected. Histologically, the IR model was shown to cause apoptosis in retinal neurons and decreased thickness in retinal cell layers, especially in the inner retinal layer [9]–[11]. Consistent with TUNEL staining results for apoptotic neurons in the inner nuclear layer, increased caspase-3 was also observed [12]. Several inflammatory and neurodegenerative processes in the retina have been observed in this animal model [5]. Increased inflammatory mediators, such as TNF-α, IL-1β, IL-6, IL-10, CCL-2 (MCP-1), CCL-5 (RANTES), CXCL-10, ICAM-1, VCAM-1, and iNOS, were noted in several studies. Notably, NF-κB is crucial for up-regulating these inflammation-associated genes [5], [11], [13]–[16].

NF (nuclear factor)-κB, a ubiquitous transcription factor, is involved in the expression of many genes associated with inflammation, cell injury and stress, and it also plays an important role in the regulation of cell survival and death. NF-κB may also be a pivotal factor in the IR injury of organs [17]–[19]. Several experimental studies have demonstrated an increased expression of activated NF-κB after retinal IR injury [13], [20], [21]. NF-κB consists of two subunits, p50 and p65 (RelA), and its activation depends on the ubiquitin-proteasome system (UPS), the major non-lysosomal pathway for intracellular protein degradation to maintain several basic cellular functions, including cell cycle progression, the stress response, and apoptosis [22]–[24]. Under normal conditions, NF-κB is bound to its inhibitory factor, IκB, and the complex is present in the cytosol. Certain stimuli, such as inflammatory signals and oxidative stress, can trigger the phosphorylation of IκB and lead to the ubiquitination and degradation of IκB by the proteasome. After dissociating from IκB, the active form of NF-κB translocates into the nucleus and promotes the transcription of related genes [25], [26].

Proteasome inhibitors have been demonstrated to be beneficial in several pathologic conditions, including autoimmune disorders in animal models and cancers in human [27], [28]. Proteasome inhibitors are also shown to have organ-protective effects in experimental IR injury of the brain, heart and kidney [29]–[32]. Blockage of NF-κB activation is thought to account for the majority of protective effects by proteasome inhibition. The effect of proteasome inhibitors on retinal IR injury has never been studied. Bortezomib (Velcade), previously known as PS-341, LDP-341 and MLM341, is a 26S proteasome inhibitor. It is approved by the FDA for use in the treatment of multiple myeloma [33]. We have demonstrated that bortezomib had anti-inflammatory effects in endotoxin-induced uveitis of rats in a previous study [34]. Concerning retinal IR injury, we hypothesized that bortezomib could inhibit the activation of NF-κB and associated inflammatory mediators, alleviate oxidative stress in the retina and reduce retinal neuron death and ganglion cell apoptosis. To provide evidence for these predictions, we designed an animal study to investigate the effect of bortezomib on pressure-induced IR injury in the rat retina.

## Methods

### Animals

We used 8-week-old, male Sprague-Dawley rats that weighed 200–250 g. The rats were injected with drugs or vehicle intraperitoneally 30 minutes prior to the induction of IR injury. The rats were randomly assigned into one of four groups:

1. The control group (sham-operated group): no drug or vehicle was given, and the anterior chamber was penetrated by needle without elevating the intraocular pressure;
2. The saline group: injection of PBS before inducing retinal ischemia;
3. The bortezomib (L) group: injection of a low dose of bortezomib (0.05 mg/kg; Millennium Pharmaceuticals, Cambridge, MA) before inducing retinal ischemia; and
4. The bortezomib (H) group: injection of a high dose of bortezomib (0.2 mg/kg) before inducing retinal ischemia.

Total number of animals used in each group of the experiments was summarized in Table 1. The treatment was blinded to the experimenter.

**Table 1:** Summary of total number of animals in each group per experiment.

Experiments	Number of animals in each group
	(n)
ERG	6
Semi-quantitative PCR	5
Western blot analysis	5
Immunofluorescence (IF) study	
iNOS	3
nitrotyrosine, 8-OHdG, acrolein	3
NF-κB p65	3
CD 68	3–4*
*in situ* TUNEL stain	3
IF stain with Neu-N	3
Fluorometric assay of proteasome activity	5

### Ethics Statement

All experiments were performed in compliance with a protocol approved by the Institutional Animal Care and Use Committee of National Taiwan University and with the ARVO Statement for the Use of Animals in Ophthalmic and Vision Research.

### Pressure-induced Ischemia-reperfusion Injury in the Retina and Tissue Retrieval

Under deep anesthesia with zolazepam+tiletamine (Zoletil 50; Virbac Laboratories, Carros, France) (25 mg/kg) and xylazine (Rompun*;* Bayer Inc., Toronto, Canada) (5 mg/kg), the anterior chamber of one eye in each rat was cannulated with a 30-gauge needle connected to a saline infusion bottle, and the bottle was elevated to a 180-cm height to achieve a 130-mmHg pressure intraocularly. The ischemic effect was confirmed by the presence of retinal blanching. The pressure was held for 45 minutes, followed by removal of the needle to regain natural reperfusion of the retina.

Some rats in each group were euthanized at 24 hours after reperfusion by intracardiac injection of phenobarbital (25 mg/kg) under deep anesthesia, and the remaining rats received a booster injection of drugs or vehicles after 3 days and were sacrificed on Day 7. The eyeballs were extracted and processed immediately for further evaluation.

### Electroretinogram (ERG)

The ERG was performed at 24 hours and 7 days after retinal reperfusion, and the rats were kept in the dark room for 24 hours before the examination. The rats were subjected to measurement of ERG waves under deep anesthesia in a room with dim light only. To normalize the data, a relative b-wave ratio indicative of the b-wave amplitude in the normal eye compared to that in the IR-injured eye of the same individual was calculated and used for statistical analysis.

### Semi-quantitative Polymerase Chain Reaction (PCR)

Twenty-four hours after the IR injury, some rats in each group were euthanized for determination of the expression of iNOS, ICAM-1, MCP-1, heme oxygenase, thioredoxin, and peroxiredoxin in the retina. Total RNA in the retina was extracted with TRIzol reagent (Invitrogen-Life Technologies, Inc., Gaithersburg, MD). One microgram of total RNA from each sample was annealed with 300 ng oligo (dT) (Promega, Madison, WI) for 5 minutes at 65°C and reverse-transcribed into cDNA using 80 U Moloney murine leukemia virus reverse transcriptase (MMLV-RT; Invitrogen-Gibco, Grand Island, NY) per 50-µg sample for 1 hour at 37°C. The reaction was halted by increasing the temperature to 90°C for 5 minutes. The cDNA product from each sample was subjected to PCR with specific primers (Table 2). The 50-µL reaction mixture contained 5 µL cDNA, 1 µL sense and antisense primers, 200 µM of each deoxynucleotide, 5 µL of 10× *Taq* polymerase buffer, and 1.25 U *Taq* polymerase (Promega). Amplification was performed in a thermocycler (MJ Research, Waltham, MA) with a 1-minute denaturation at 94°C and a 3-minute extension at 72°C. The annealing temperature was between 62°C and 42°C, and the temperature was decreased in 1°C increments, followed by 21 cycles at 55°C. Finally, the temperature was elevated to 72°C for 10 minutes and then reduced to 4°C. We obtained a 10-µL sample of each PCR product to perform electrophoresis on 2% agarose gels containing ethidium bromide (Sigma-Aldrich). The results were analyzed under ultra-violet light using DNA molecular length markers. The intensity was quantified using image analysis software (Photoshop, ver.7.0; Adobe Systems, San Jose, CA), and the results were standardized against the intensity of rat β-actin, a housekeeping gene.

**Table 2:** Primer sequences for iNOS, MCP-1, ICAM-1, and anti-oxidant proteins for semi-quantitative PCR.

Genes	Primer sequences	Product size
		(bp)
**iNOS**	5′-TATCTGCAGACACATACTTTACGC-3′ 5′-TCCTGGAACCACTCGTACTTG-3′	344
**MCP-1**	5′-CTGGGCCTGTTGTTCACAGTTGC-3′ 5′-CTACAGAAGTGCTTGAGGTGGTTG-3′	436
**ICAM-1**	5′-CCTGTTTCCTGCCTCTGAAG-3′ 5′-CCTGGGGGAAGTACTGTTCA-3′	830
**Heme**	5′-CACGCCTACACCCGCTACCT-3′	209
**oxygenase-1**	5′-TCTGTCACCCTGTGCTTGAC-3′	
**Thioredoxin**	5′-CTGCTTTTCAGGAAGCCTTG-3′ 5′-TGTTGGCATGCATTTGACTT-3′	203
**Peroxiredoxin**	5′-TGCCAGATGGTCAGTTTAAA-3′ 5′-CAGCTGGGCACACTTCCCCA-3′	465
**β-actin**	5′-CTGGAGAAGAGCTATGAGCTG-3′ 5′-AATCTCCTTCTGCATCCTGTC-3′	246

### Western Blot Analysis

For the rats sacrificed at 24 hours after retinal reperfusion, total protein was extracted from the retina by lysing the sample in radioimmunoprecipitation assay (RIPA) buffer [0.5 M Tris-HCl (pH 7.4), 1.5 M NaCl, 2.5% deoxycholic acid, 10% NP-40, 10 mM EDTA and protease inhibitors (Complete Mini; Roche Diagnostics Corp., Indianapolis, IN)]. The extract and Laemmli buffer were mixed at a 1∶1 ratio, and the mixture was boiled for 5 minutes. A 100-µg sample was separated on 10% SDS-polyacrylamide gels and then transferred to polyvinylidene difluoride membranes (Immobilon-P; Millipore Corp., Billerica, MA). The membranes were incubated with anti-iNOS, anti-ICAM-1, anti-TNF-α, anti-p53, anti-bax, and anti-β-actin antibodies. Then, the membranes were incubated with horseradish peroxidase-conjugated secondary antibody and visualized by chemiluminescence (GE Healthcare). The density of blots was determined using image-analysis software after scanning the image (Photoshop, ver.7.0; Adobe Systems, San Jose, CA). The optical densities of each band were calculated and standardized based on the density of the β-actin band.

### Immunofluorescence (IF) Stain of the Retina

Twenty-four hours after IR injury of the retina, the eyeballs were enucleated and immersed in 4% paraformaldehyde in 0.2 M phosphate buffer for 24 hours. After fixation, the eyes were dehydrated with alcohol and then embedded in paraffin. The specimens were cut into 5-µm sagittal sections near the optic nerve head to evaluate the expression of iNOS, nitrotyrosine, 8-OHdG, acrolein, NF-κB p65, and CD 68 in the retina.

After deparaffinization with xylene solutions and rehydration with a graded series of ethanol in PBS, the tissue sections were incubated overnight with monoclonal antibodies against iNOS, nitrotyrosine, 8-OHdG, acrolein, CD 68 (Santa Cruz Biotechnology, Santa Cruz, CA) and the p65 subunit of NF-κB (Chemicon, Temecula, CA) separately at 4°C. The immunoreactivity was detected by adding a rhodamine-labeled (for CD 68) or fluorescein isothiocyanate (FITC)-labeled (for the others) secondary antibody (Abcam, Cambridge, U.K.), and the cell nuclei were counterstained with 4′,6-diamidino-2-phenylindole (DAPI). Specimens stained without the primary antibody were used as negative controls.

### *In situ* TdT-mediated dUTP Nick-end Labeling (TUNEL) Assay

Retinal cell apoptosis was determined by TUNEL assay at 24 hours after the IR injury. The retinal sections were stained using a TUNEL-based kit (TdT FragEL; Oncogene, Darmstadt, Germany) according to the manufacturer’s instructions. The sections incubated with DNase I prior to the labeling procedure were used as positive controls, and the sections stained with label solution containing no terminal transferase were used as negative controls.

### NeuN Stain in Flat-mounted Retina and Counting of NeuN-positive Cells

The density of retinal ganglion cells (RGCs) was evaluated by immunofluorescence staining with NeuN 7 days after retinal reperfusion. Briefly, the eyeballs were incised at the ora serrata and immersion-fixed in a 4% paraformaldehyde solution for 1 h, and the retina tissues were extracted. The retina was cryoprotected overnight in 30% sucrose followed by three freeze–thaw cycles and incubated overnight with monoclonal FITC-conjugated NeuN antibody (Chemicon, USA). Finally, the retina was flat-mounted and viewed with a Leica TSL AOBS SP5 confocal microscope (Leica Microsystems, Exton, PA, USA).

The number of NeuN-positive cells for each retinal section was counted in 4 selected retinal areas located at the same eccentricity (approximately 1.5 mm from the optic disk) in the four retinal quadrants. The cell number was quantified with image-analysis software (Image-Pro Plus, ver.6.0; Mediacybernetic, Atlanta, GA, USA).

### Measurement of Proteasome Activity in the Retina

The chymotrypsin-like activity of the proteasome in the rats sacrificed at 24 hours after retinal reperfusion was determined using commercial proteassome assay kits (Proteasome-Glo™ assay systems; Promega) according to the manufacturer’s instructions. Briefly, the Suc-LLVY-Glo™ substrate was added to the mixture of the Proteasome-Glo™ buffer and the luciferin detection reagent and incubated at room temperature for 1 hour. The retinal tissue was minced in 100 µL of ice-cold buffer PBS containing 5 mM EDTA followed by centrifugation at 12000 g at 4°C for 10 minutes. A 50-µL retinal sample was added by equal volume of reagent mixture and incubated for 1.5 hours. Finally, the luminescence of retinal sample was detected by a microplate luminometer (Promega).

### Statistical Analysis

The values are shown as the mean ± SD. To compare the numerical data among the four groups, Kruskal Wallis H test followed by post hoc Dunn test was used. *P*<0.05 was considered statistically significant.

## Results

### The Effect of Bortezomib on Retinal Functional Change following IR Injury

**Figure 1:**
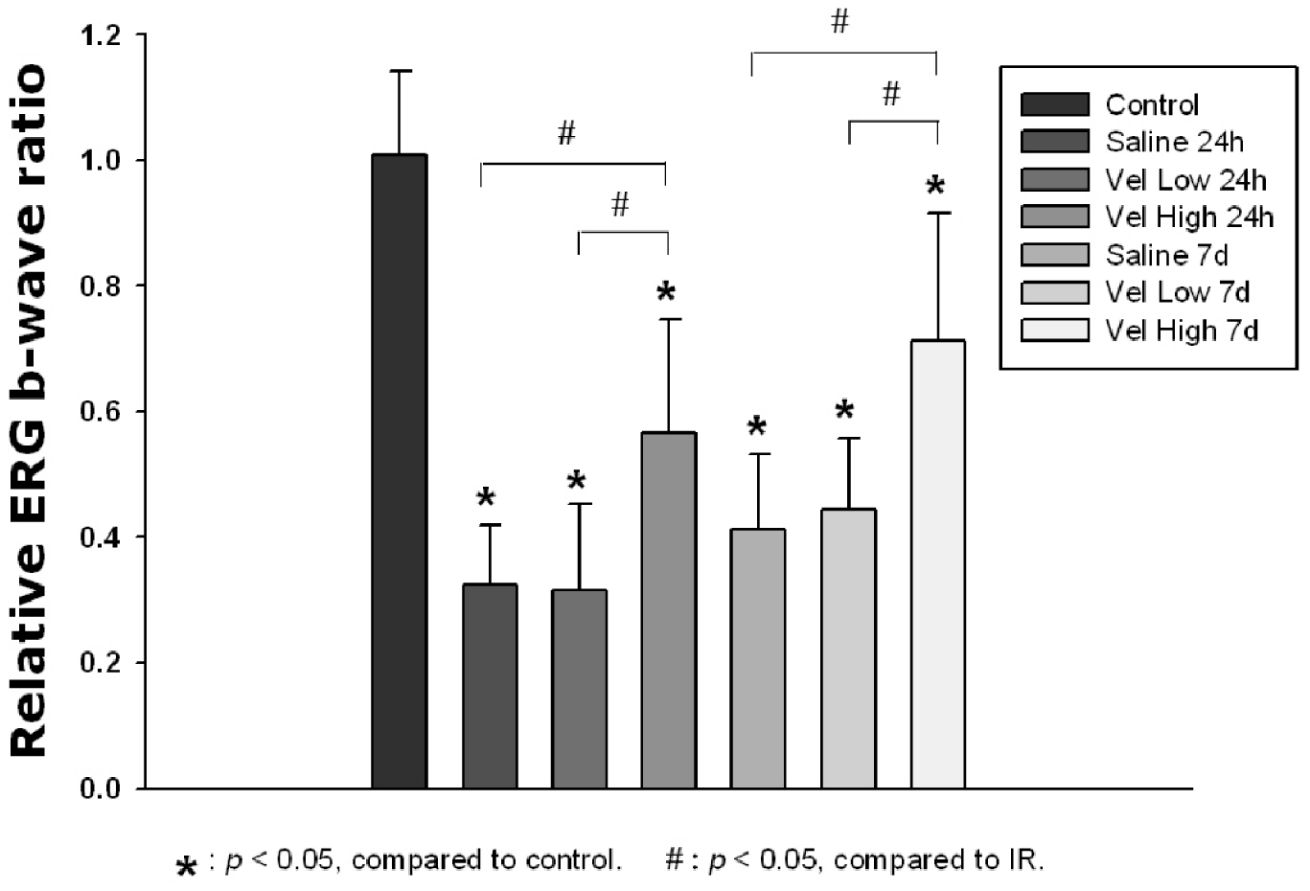
Evaluation of functional changes of the retina by ERG. The relative b-wave ratio was significantly decreased in the saline, low-dose bortezomib [Vel (L)] and high-dose bortezomib [Vel (H)] groups compared with the control group, both at 24 hours and 7 days after injury. Notably, the relative b-wave ratio in the high-dose bortezomib [Vel (H)] group was significantly higher than that in the saline and low-dose bortezomib [Vel (L)] groups in time-matched comparisons. The data are expressed as the mean ± SD of the mean in 6 rats for each group (bar graph). *P<0.05 compared with the control group. ^#^ *P*<0.05 by Kruskal Wallis H test with post hoc Dunn test.

The relative b-wave ratio was approximately equal to 1 in the normal controls. For the IR-injured rats, whether pretreated with saline, low-dose bortezomib or high-dose bortezomib, the ratios were all significantly decreased, both at 24 hours and 7 days after injury (*P*<0.05 in all paired comparisons with normal rats). Notably, in rats pretreated with high-dose bortezomib, the relative b-wave ratio was higher than that in the saline and the low-dose bortezomib groups, both at 24 hours (*P* = 0.001 and *P* = 0.002) and 7 days (*P* = 0.021 and *P* = 0.014) after the IR injury (Figure 1).

### The Effect of Bortezomib on the mRNA Expression of Inflammatory Mediators and Anti-oxidant Proteins in IR-injured Retinas

**Figure 2:**
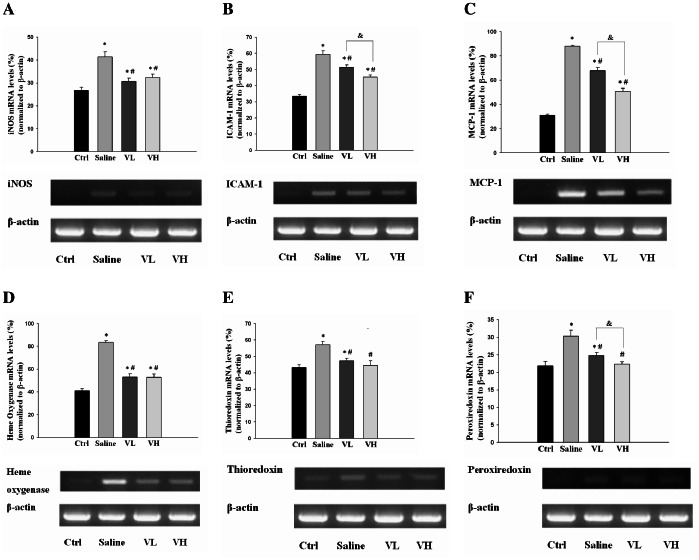
Evaluation of the mRNA expression of inflammatory mediators and anti-oxidant proteins by PCR. The mRNA expression levels of iNOS (A), ICAM-1 (B) and MCP-1 (C) were significantly higher in the saline, low-dose bortezomib [Vel (L)] and high-dose bortezomib [Vel (H)] groups compared with normal rats. The expression of these inflammatory mediators was significantly lower in the bortezomib-pretreated groups, especially in the high-dose group, than in the saline group. The mRNA expression levels of heme oxygenase (C), thioredoxin (D) and peroxiredoxin (E) were significantly higher in the saline group compared with the control group. The expression of these anti-oxidant proteins was significantly lower in the bortezomib-pretreated groups than in the saline group, and the levels of thioredoxin and peroxiredoxin did not differ significantly between the high-dose bortezomib and control groups. The data are expressed as the mean ± SD of the mean in 5 rats for each group (bar graph). *P<0.05 compared with the control group. ^#^ *P*<0.05 compared with the saline group. & *P*<0.05 by Kruskal Wallis H test with post hoc Dunn test.

The mRNA expression levels of iNOS, ICAM-1 and MCP-1 were significantly higher in the IR-injured rats pretreated with saline compared with normal rats (*P*<0.05 in all paired comparisons). In the IR-injured rats pretreated with low- or high-dose bortezomib, the expression of these inflammatory mediators was significantly lower than that in the saline group (*P*<0.05 in all paired comparisons). In addition, the levels of ICAM-1 and MCP-1 were more reduced in the high-dose bortezomib group than in the low-dose bortezomib group (*P* = 0.004 and <0.001, respectively) (Figure 2A, 2B, 2C).

In a similar fashion, the mRNA expression levels of heme oxygenase, thioredoxin and peroxiredoxin were significantly higher in the saline group compared with the control group (*P*<0.05 in all paired comparisons). The expression of these anti-oxidant proteins was significantly lower in the bortezomib groups than in the saline group (*P*<0.05 in all paired comparisons), and the levels of thioredoxin and peroxiredoxin did not differ significantly between the high-dose bortezomib and control groups (*P* = 0.900 and 0.970, respectively) (Figure 2D, 2E, 2F).

### The Influence of Bortezomib on the Levels of Inflammatory Mediators and Pro-apoptotic Proteins in IR-injured Retinas

**Figure 3:**
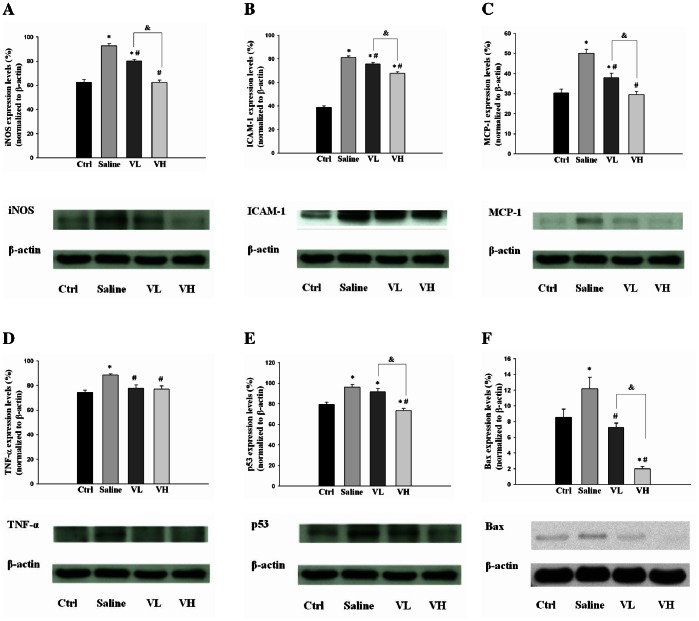
Evaluation of the expression of inflammatory mediators and pro-apoptotic proteins by western blot analysis. The protein levels of iNOS (A), ICAM-1 (B), MCP-1 (C) and TNF-α (D) were significantly higher in the saline group compared with normal rats. In the bortezomib-pretreated groups, especially in the high-dose group, the levels of inflammatory mediators were significantly lower than in the saline group. The levels of p53 (E) and bax (F) were significantly higher in the saline group compared with the control group. The expression levels of p53 and bax were lower in the bortezomib-pretreated groups, especially in the high-dose group, compared with the saline group. The data are expressed as the mean ± SD of the mean in 5 rats for each group (bar graph). *P<0.05 compared with the control group. ^#^ *P*<0.05 compared with the saline group. & *P*<0.05 by Kruskal Wallis H test with post hoc Dunn test.

The protein levels of iNOS, ICAM-1, MCP-1 and TNF-α were significantly higher in the IR-injured rats pretreated with saline compared with normal rats (*P*<0.001 in all paired comparisons). In the IR-injured rats pretreated with low- or high-dose bortezomib, these inflammatory mediators were significantly lower than in the saline group (*P*<0.05 in all paired comparisons). In addition, the levels of iNOS, ICAM-1 and MCP-1 were more reduced in the high-dose bortezomib group than in the low-dose bortezomib group (*P*<0.05 in all paired comparisons) (Figure 3A, 3B, 3C and 3D).

The levels of p53 and bax were significantly higher in the saline group compared with the control group (*P*<0.05 in all paired comparisons). The expression levels of p53 and bax were lower in the bortezomib group compared with the saline group, and the differences were statistically significant in the high-dose bortezomib group (*P*<0.001 in all paired comparisons) (Figure 3E and 3F).

### The Effect of Bortezomib on the Expression of iNOS and Oxidative Markers in IR-injured Retinas

**Figure 4:**
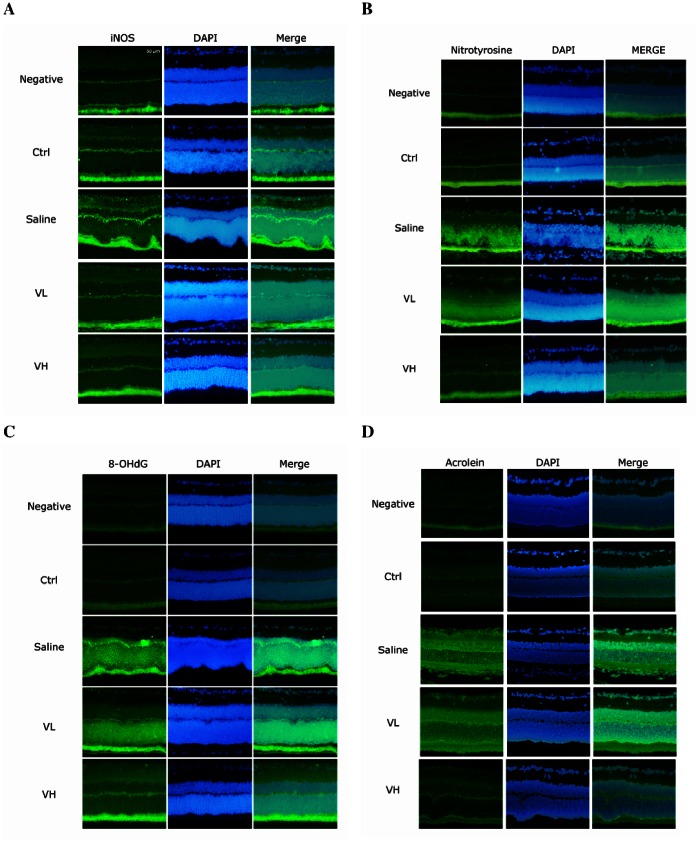
Evaluation of the expression of iNOS and oxidative markers in the retina using IF staining. There was marked expression of iNOS (A) in each retinal layer in the saline group compared with the control group. In the bortezomib-pretreated groups, the expression of iNOS was less prominent, especially in the high-dose bortezomib [Vel (H)] group. Increased staining of nitrotyrosine (B), 8-OHdG (C), and acrolein (D) was noted in the saline group compared with the control group, and expression of these oxidative markers were less prominent in the bortezomib-pretreated groups, especially in the high-dose bortezomib group [Vel (H)]. The images represent three rats in each group. There was little variation between eyes in the same group.

IF staining revealed marked expression of iNOS in each retinal layer in the saline group compared with the control group. In rats pretreated with bortezomib, the expression of iNOS was less prominent, especially in the high-dose bortezomib group (Figure 4A).

In a similar fashion, increased expression levels of nitrotyrosine, 8-OHdG, and acrolein in the retina were noted in the saline group compared with the control group, and the expression levels of these oxidative markers were less pronounced in the bortezomib groups, especially in the high-dose bortezomib group (Figure 4B, 4C and 4D).

### The Inhibitory Effect of Bortezomib on NF-κB Activation in Retinal IR Injury

**Figure 5:**
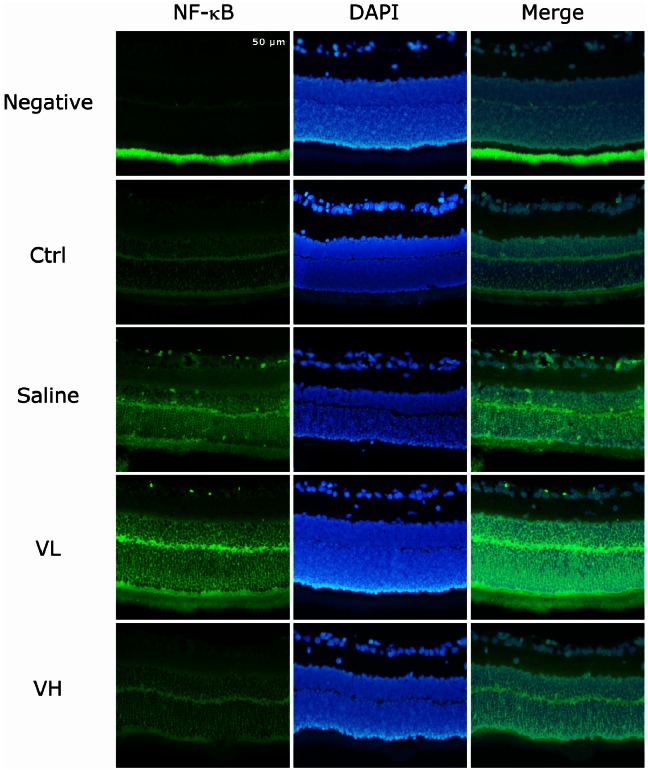
IF study for evaluating the expression of NF-κB p65 in the retina. There was increased staining of the NF-κB p65 subunit in the retinal layer in the saline group and the low-dose bortezomib group [Vel (L)] but not in the high-dose bortezomib group [Vel (H)]. The images represent three rats in each group. There was little variation between eyes in the same group.

Increased staining of the NF-κB p65 subunit in each layer of the retina was noted in the saline group and low-dose bortezomib group. In contrast, there was no significant difference in p65 expression between the high-dose bortezomib group and the control group (Figure 5).

### Bortezomib Reduced the Recruitment of CD 68 Cells in IR-injured Retinas

**Figure 6:**
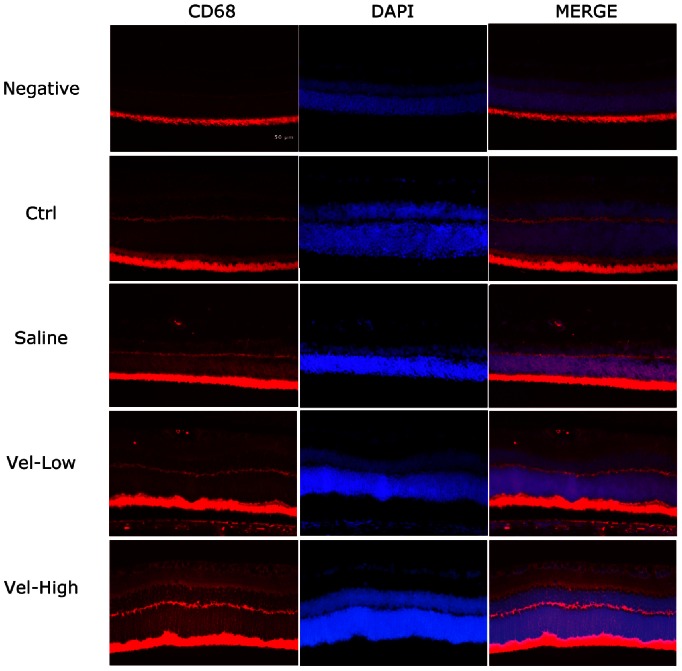
IF study for evaluating CD 68-positive cells infiltrating the retina. No CD 68-positive cells were found in the control group. Some CD 68 cells were noted in the inner retina in the saline group, as well as in the low-dose bortezomib group [Vel (L)]. However, no CD 68 cells were found in the high-dose bortezomib group [Vel (H)]. The images represent three rats in each group. There was little variation between eyes in the same group.

Normally, nearly no CD 68-positive cells were present in the retina. Some CD 68 cells were noted in the inner retinal tissue in the saline group, as well as in the low-dose bortezomib group. However, in the high-dose bortezomib group, no CD 68 cells were found in the retinal sections (Figure 6).

### The Anti-apoptotic Effect of Bortezomib on IR-injured Retinas

**Figure 7:**
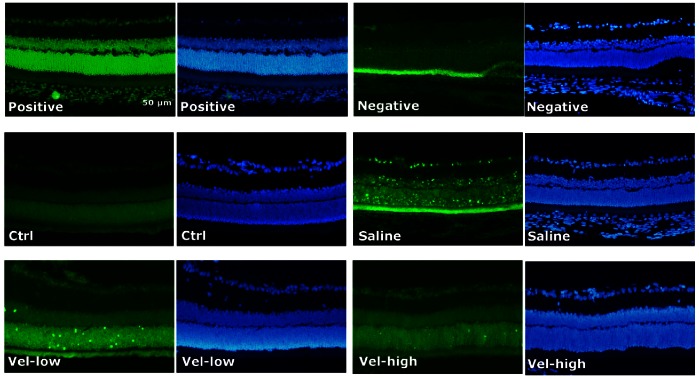
Evaluation of cell apoptosis in the retina with *in situ* TUNEL staining. TUNEL-positive nuclei in each layer of retinal tissue were noted in the saline group but not in the control group (Ctrl). In the bortezomib-pretreated groups, especially in the high-dose group (Vel-High), the density of TUNEL-positive cells was markedly reduced compared with the saline group. The images represent three rats in each group. There was little variation between eyes in the same group. Positive: positive controls: tissue sections incubated with DNase I prior to the labeling procedure. Negative: negative controls: tissue sections stained with label solution containing no terminal transferase.

Increased cell apoptosis in each retinal layer was noted in the saline group compared with the control group by *in situ* TUNEL staining. In the IR-injured rats pretreated with bortezomib, especially in the high dosage group, the density of TUNEL-positive cells was markedly reduced compared with rats pretreated with saline (Figure 7).

### The Influence of Bortezomib on the Density of Retinal Ganglion Cells in IR-injured Retinas

**Figure 8:**
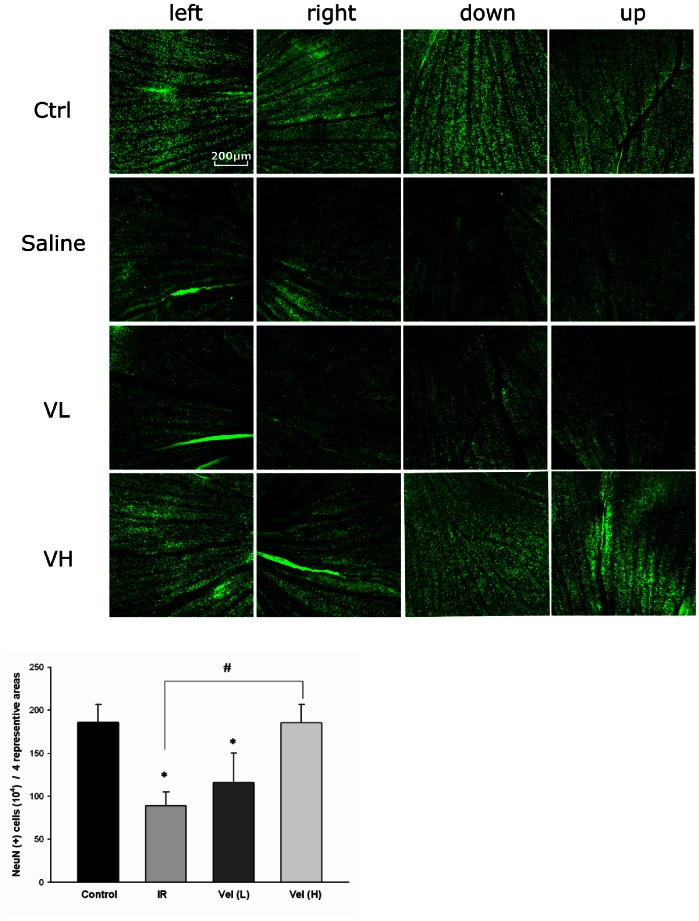
Evaluation and quantification of retinal ganglion cells assisted with Neu-N staining. Compared with the control group, there were significantly decreased numbers of retinal ganglion cells in the saline and the low-dose bortezomib [Vel (L)] groups but not in the high-dose bortezomib group [Vel (H)]. The images represent three rats in each group. There was little variation between eyes in the same group. The data are expressed as the mean ± SD of the mean in 3 rats for each group (bar graph). *P<0.05 compared with the control group. ^#^ *P*<0.05 by Kruskal Wallis H test with post hoc Dunn test.

Decreased numbers of retinal ganglion cells were noted in the saline and the low-dose bortezomib groups compared with those in the control group by Neu-N IF staining (*P* = 0.005 and 0.028, respectively). However, there was no difference in the numbers of retinal ganglion cells between the high-dose bortezomib group and the control group (*P* = 0.989) (Figure 8).

### The Effect of Bortezomib on Chymotrypsin-like Activity of the Proteasome in IR-injured Retinas

**Figure 9:**
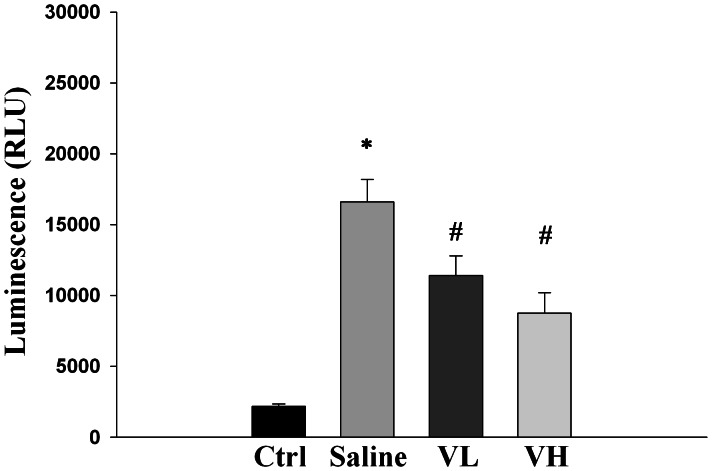
Evaluation of chymotrypsin-like activity of the proteasome by fluorometric measurement. Compared with the control group, there was significantly increased signal of luminescence in the saline, the low-dose [Vel (L)], and the high-dose bortezomib [Vel (H)] groups. The signals were markedly lowered in the bortezomib-pretreated groups compared with the saline group. No statistically significant difference was observed between the low-dose and the high-dose bortezomib groups. The data are expressed as the mean ± SD of the mean in 5 rats for each group (bar graph). *P<0.05 compared with the control group. ^#^ *P*<0.05 by Kruskal Wallis H test with post hoc Dunn test.

Increased signal of luminescence, indicating increased chymotrypsin-like activity, in the retinal tissues was noted in the saline group compared with the control group. The signals were significantly lower in the bortezomib-pretreated groups than in the saline group. There was no significance difference in the signal of luminescence between the low-dose and the high-dose bortezomib groups (*P* = 0.125) (Figure 9).

There was no mortality or extraocular morbidity associated with the induction of IR injury or the treatment in the experimental animals. The body weight and the number of the red blood cell (RBC) and white blood cell (WBC) of the rats did not differ significantly between the control and the treated groups both at 24 hours and on Day 7 (Figure S1, S2).

## Discussion

Our study demonstrated that pretreatment with bortezomib could dose-dependently reduce the b-wave decrement in ERG, and PCR and western blot analysis showed that pretreatment with bortezomib could decrease the expression of inflammatory mediators and production of p53 and bax. IF staining of retinal sections revealed that bortezomib decreased tissue oxidative stress, activation of NF-κB and recruitment of CD 68 cells resulting from IR insult. We observed that cell apoptosis, revealed by *in situ* TUNEL staining, was attenuated by bortezomib. IF staining with Neu-N indicated that retinal ganglion cells were largely preserved in the high-dose bortezomib group after 7 days of initiating IR injury.

Currently, the clinical application of bortezomib is primarily in the treatment of human hematologic diseases, such as multiple myeloma, mantle cell lymphoma, and acute graft-versus-host disease after allograft bone marrow transplantation [35], [36]. Other potential applications of bortezomib, such as the anti-inflammatory effects in autoimmune diseases and the protective effects in IR injury of brain, heart, and kidney [27]–[32], [34], [37], [38], are under investigation and currently limited to use in animal models. Although not fully understood, the postulated mechanism of the drug’s effect is primarily achieved through the inhibition of NF-κB activation, which in turn reduces the transcription of inflammation-related genes and avoids the induction of inflammatory cascades. Therefore, a proteasome inhibitor could protect target tissues from inflammation-induced oxidative stress and cell damage.

Whether proteasome function is altered in the process of IR injury is still controversial. Increased proteasome activity has been observed in an experimental study of IR injury in liver tissue, as well as in the retina demonstrated by our study [39]. Moreover, proteasome inhibitors theoretically normalize the proteasome function and exert anti-inflammatory effects through the inactivation of NF-κB. However, impaired proteasome function has been found in some animal studies of IR injury in brain and heart tissue. A plausible explanation for this impairment is the depletion of ATP during ischemia, which inhibits the conversion of 20S to 26S proteasome. Another possibility is that the oxidative protein and lipid products interfere with the enzyme activity of proteasomes. Furthermore, the functional impairment of proteasomes will lead to the intracellular accumulation of oxidative and ubiquitinated proteins, resulting in a vicious cycle [40]–[42]. Paradoxically, the use of proteasome inhibitors in these animal models has been demonstrated to lead to a tissue-protective effect, which may be related to the time of drug administration. It has been postulated that short-term use of proteasome inhibitors in the acute stage of IR injury, during which the proteasome function was only minimally affected, may be more beneficial than persistent use of the drug in the chronic stage [32].

Our study demonstrated that the activation of NF-κB was involved in the pathogenesis of IR injury in the retina, which was compatible with the results of other animal studies [11], [13], [20], [21]. In these studies, the activation of NF-κB was found to peak at 12 to 24 hours after the injury [20], [21]. Furthermore, several studies have demonstrated that the activation of NF-κB was associated with an increased expression of inflammatory mediators, including TNF-α, MCP-1 (CCL2), ICAM-1, VCAM-1, and iNOS, after retinal IR injury. These studies also showed that inhibition of NF-κB could reduce the cell damage [11], [13]–[16], [43]. Increased levels of TNF-α were observed in the early stage of retinal ischemia and in several neurodegenerative diseases, and TNF-α was proposed as a pivotal cytokine mediating neuron death [44]–[47]. MCP-1 is an important chemokine in attracting monocytes/macrophages to target tissues [48]. TNF-α is one of the cytokines responsible for up-regulation of ICAM-1 expression in endothelial cells of blood vessels to facilitate leukocyte adhesion and transmigration into interstitial space [49]–[51]. As a result, the increase of inflammatory cells can lead to occlusion of the capillaries in local tissue, which further augments the ischemic insult. Further oxidative products are generated, and severe tissue damage ensues [52]. In addition, increased expression of iNOS in infiltrating leukocytes and inner retinal layer occurs after retinal ischemia, and the increased production of NO, a cytotoxic free radical, results in neural cell death by impairing oxidative enzyme activity, leading to the formation of oxidative products, and directly attacking DNA [44], [53]–[55]. Our experiment demonstrated that bortezomib could inhibit the NF-κB activation after retinal IR injury and decrease the expression of TNF-α, MCP-1, ICAM-1 and iNOS. Bortezomib also inhibited the infiltration of CD 68 (a cell marker of microglia/macrophages)-positive cells and decreased the expression of oxidative markers, including nitrotyrosine, 8-OHdG and acolein, in retinal tissue. Decreased production of anti-oxidant proteins, including heme oxygenase-1, thioredoxin, and peroxiredoxin, presumably occurred because the drug decreased oxidative stress with less induction of these anti-oxidant proteins.

Several studies indicated that oxidative stress is one of the most important causes of retinal neuron death after IR injury and demonstrated that the damage can be alleviated by decreasing oxidative stress [56]–[59]. Decreasing the production of free radicals could reduce the activation of NF-κB and down-regulate the expression of TNF-α, which is the ligand for the TNFR-1/Fas-mediated extrinsic pathway of apoptosis [60]. In addition, the activated NF-κB could promote cell apoptosis by up-regulating p53 expression and interrupting the balance between bax and bcl-xL/bcl-2 [61]–[64]. Therefore, inhibition of NF-κB may protect cells from apoptosis by decreasing p53 expression. Our study demonstrated that bortezomib could reduce the inflammation response and oxidative stress after retinal IR injury. In addition, the expression of p53 and bax was reduced, retinal cell apoptosis was decreased and retinal function was preserved.

In the present study, the results showed that bortezomib had protective effects in retinal IR injury, both anatomically and functionally. No optimal treatment methods yet exist for several sight-threatening ophthalmic disorders involving the mechanisms of IR injury, and proteasome inhibition may be a potential strategy for managing these diseases. Our study has some limitations. First, bortezomib was administered before the induction of ischemia to ensure the onset of the drug’s effect, which would not happen in a clinical situation. However, the main purpose of our study was to evaluate the drug’s effect and possible mechanisms, so we still consider the results to be informative and referable. Second, due to the ubiquitous distribution of proteasomes, the systemic administration of proteasome inhibitors inevitably causes many unwanted adverse effects. Therefore, investigation of the heterogeneity between proteasomes in different organs is mandatory. These differences include the composition of subunits, protein structures, post-translational modification, and the associating partners of proteasomes [32], [65]. The designation of organ-specific proteasome inhibitors theoretically provides better efficacy with increased safety. In conclusion, our study demonstrated that bortezomib successfully inhibited the activation of NF-κB and the induction of inflammatory cascades after retinal IR injury in a dose-dependent manner. Administration of bortezomib also reduced the oxidative stress in tissues and exerted protective effects on retinal tissue, both anatomically and functionally.

## Supporting Information

Figure S1 **Body weight of the rats in different groups.** The body weight of the rats didn’t differ significantly between the control and the treated groups both at 24 hours (A) and on Day 7 (B). The data are expressed as the mean ± SD of the mean in 3 rats for each group (bar graph). Statistical analysis by Kruskal Wallis H test with post hoc Dunn test.
(TIF)

Figure S2 **The blood count of the rats in different groups.** There was no statistically significant difference in the RBC count between the control and the treated groups both at 24 hours (A) and on Day 7 (B). Similarly, no statistically significant difference in WBC count was noted between the four groups both at 24 hours (C) and on Day 7 (D). The data are expressed as the mean ± SD of the mean in 3 rats for each group (bar graph). Statistical analysis by Kruskal Wallis H test with post hoc Dunn test.
(TIF)
