# Supplementary figures and images for: The Protective Effects of the Proteasome Inhibitor Bortezomib (Velcade) on Ischemia-Reperfusion Injury in the Rat Retina
Source: PLoS One. 2013 May 14;8(5):e64262. doi: 10.1371/journal.pone.0064262 (PMC3653862; doi:10.1371/journal.pone.0064262)

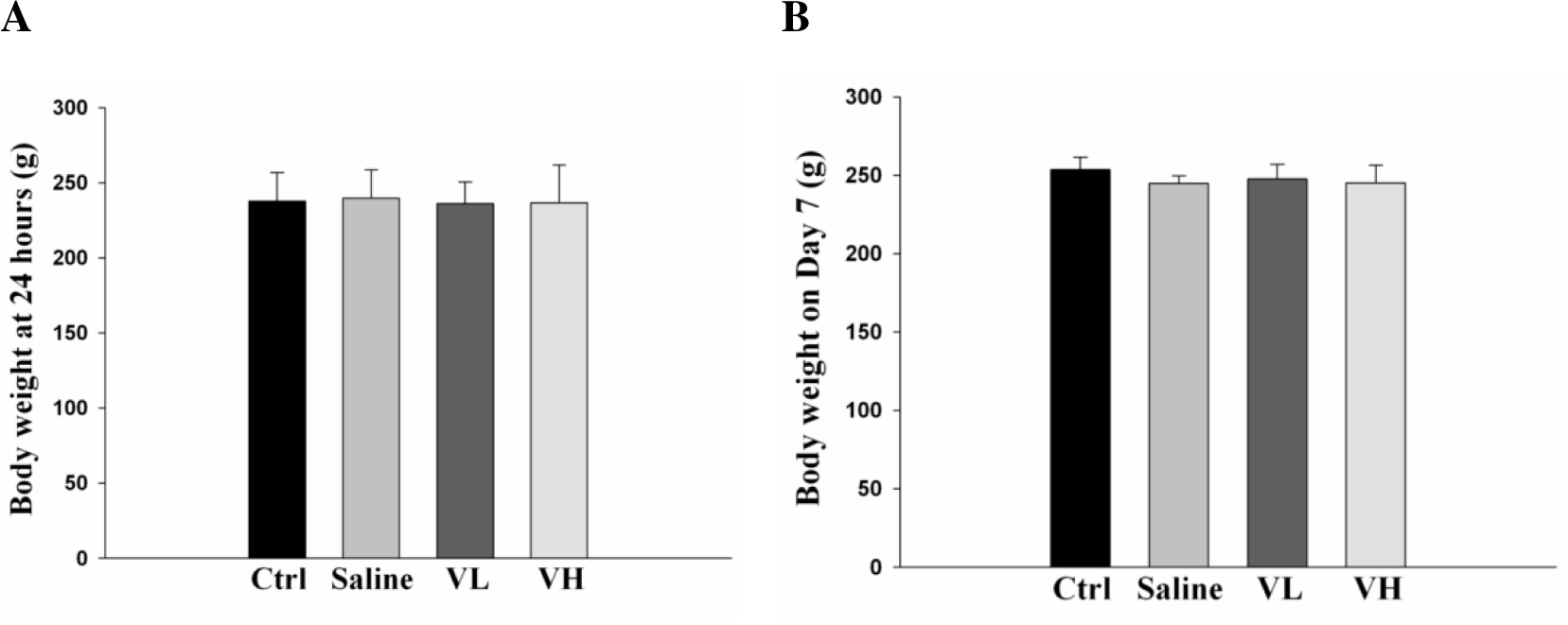

Supplement: Figure S1 — Body weight of the rats in different groups. The body weight of the rats didn’t differ significantly between the control and the treated groups both at 24 hours (A) and on Day 7 (B). The data are expressed as the mean ± SD of the mean in 3 rats for each group (bar graph). Statistical analysis by Kruskal Wallis H test with post hoc Dunn test. (TIF) [file pone.0064262.s001.tif]

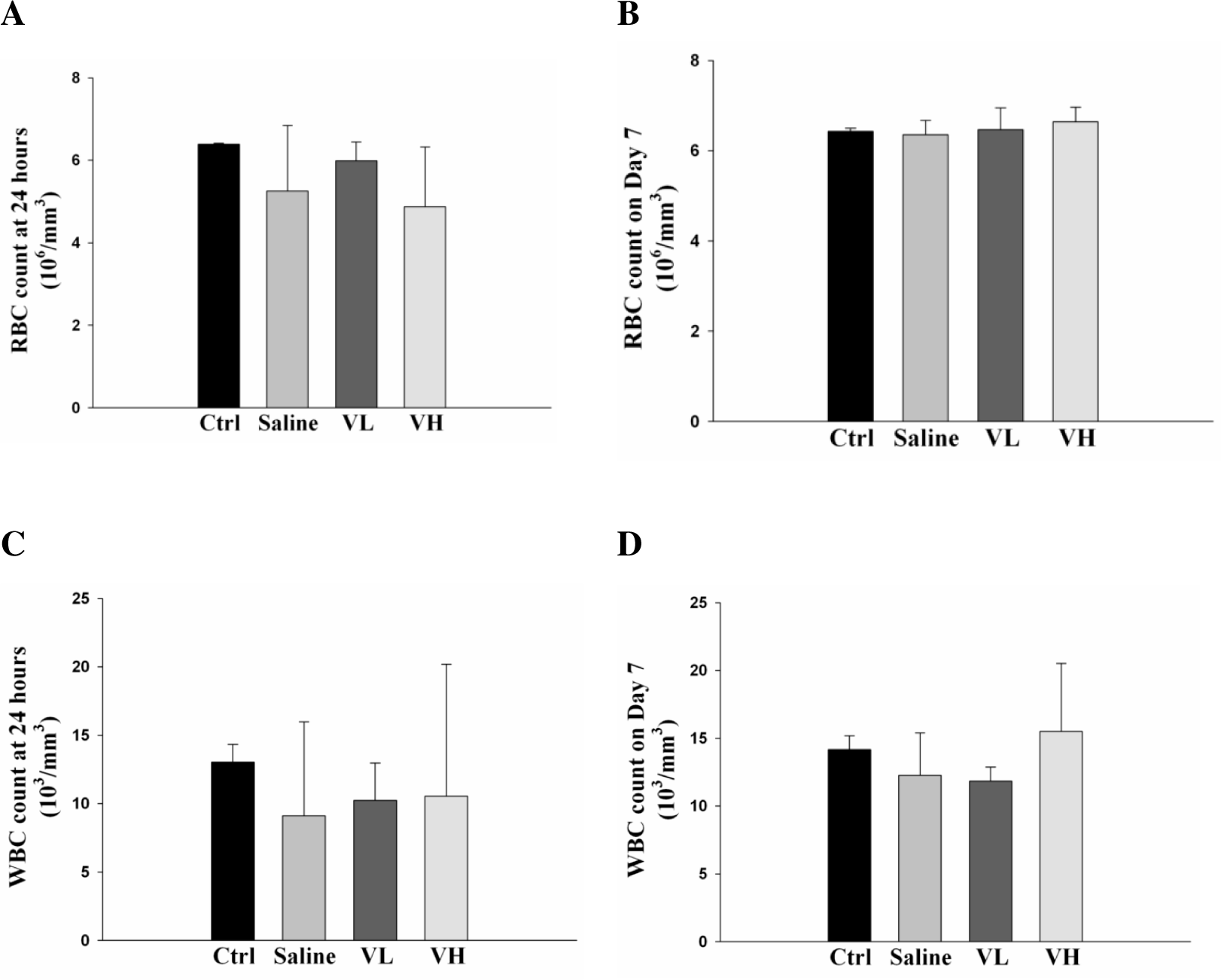

Supplement: Figure S2 — The blood count of the rats in different groups. There was no statistically significant difference in the RBC count between the control and the treated groups both at 24 hours (A) and on Day 7 (B). Similarly, no statistically significant difference in WBC count was noted between the four groups both at 24 hours (C) and on Day 7 (D). The data are expressed as the mean ± SD of the mean in 3 rats for each group (bar graph). Statistical analysis by Kruskal Wallis H test with post hoc Dunn test. (TIF) [file pone.0064262.s002.tif]
